# This Number of the Journal

**Published:** 1881-01

**Authors:** 


This Number of the Journal will be sent to a few carefully selected addresses among the most prominent and reputable physicians and dentists who are not yet upon our subscribers list, but whom we hope will be induced to permit the enrollment of their names for the ensuing year ? This issue goes forth as an earnest of what our readers may expect in the succeeding ones.

